# Transplantation of bone marrow-derived mesenchymal stem cells (BMSCs) improves brain ischemia-induced pulmonary injury in rats associated to TNF-α expression

**DOI:** 10.1186/s12993-016-0093-0

**Published:** 2016-03-01

**Authors:** Qin-qin He, Xiang He, Yan-ping Wang, Yu Zou, Qing-jie Xia, Liu-Lin Xiong, Chao-zhi Luo, Xiao-song Hu, Jia Liu, Ting-hua Wang

**Affiliations:** Department of Anesthesia and Critical Care Medicine Translational Neuroscience Center, West China Hospital, Sichuan University, Chengdu, 610041 Sichuan China; Institute of Neuroscience and Experiment Animal Center, Kunming Medical University, Kunming, 650031 China; Center for Experimental Technology for Preclinical Medicine, Chengdu Medical College, Chengdu, 610083 Sichuan China

**Keywords:** Brain ischemia, Acute lung injury, Bone marrow mesenchymal stem cells, TNF-α

## Abstract

**Background:**

Bone marrow mesenchymal stem cell (BMSCs)-based therapy seems to be a promising treatment for acute lung injury, but the therapeutic effects of BMSCs transplantation on acute lung injury induced by brain ischemia and the mechanisms have not been totally elucidated. This study explores the effects of transplantation of BMSCs on acute lung injury induced by focal cerebral ischemia and investigates the underlying mechanism.

**Methods:**

Acute lung injury model was induced by middle cerebral artery occlusion (MCAO). BMSCs (with concentration of 1 × 10^6^/ml) were transplanted into host through tail vein 1 day after MCAO. Then, the survival, proliferation and migration of BMSCs in lung were observed at 4 days after transplantation, and histology observation and lung function were assessed for 7 days. Meanwhile, in situ hybridization (ISH), qRT-PCR and western blotting were employed to detect the expression of TNF-α in lung.

**Results:**

Neurobehavioral deficits and acute lung injury could be seen in brain ischemia rats. Implanted BMSCs could survive in the lung, and relieve pulmonary edema, improve lung function, as well as down regulate TNF-α expression.

**Conclusions:**

The grafted BMSCs can survive and migrate widespread in lung and ameliorate lung injury induced by focal cerebral ischemia in the MCAO rat models. The underlying molecular mechanism, at least partially, is related to the suppression of TNF-α.

## Background

Over the past decades in the United States, the relative rate of stroke death has fallen and the actual number of stroke deaths has declined from the third to the fourth leading cause of death in population suffered stroke [1]. However, in low- and middle- income countries, the incidence and mortality of stroke were still disproportionately high [2], in which, about 95,000 people still experience new or recurrent stroke (ischemic or hemorrhagic). After stroke, several complications are still great challenges to physicians. Among them, not only is the presence of pulmonary dysfunction after stroke well recognized, but also brain lung crosstalk as a complex interaction, has been recognized [3–7]. Severe pulmonary injuries occurred not only in stroke condition, but also induced in brain injuries, such as severe traumatic brain injury (TBI) or subarachnoid hemorrhage (SAH) [8–11]. As pulmonary dysfunction, such as pulmonary edema (NPE) [6, 12], pneumonia [7], acute lung injury and the acute respiratory distress syndrome (ALI/ARDS) [9], is severe and often result in the increase of mortality, or lead to the poor neurological outcome and longer intensive care unit (ICU) and longer length of hospital stay after stroke [8, 9, 13]. Therefore, it is very important to find the effective method for the treatment of lung injury, and investigate the molecular mechanism for find the intervention strategy.

Currently, the pathophysiology of lung dysfunction after stroke is still in debate, with several theories proposed. Theodore and Robin et al. first defined the “blast theory” of neurogenic pulmonary edema (NPE). In the blast theory, transient increase of intravascular pressure, caused by an acute increase in intracranial pressure (ICP), damages the capillary-alveolar membranes which may cause a leak of protein-rich plasma [14]. However, continuous hemodynamic monitoring indicated that NPE may be irrelevant with hemodynamic instability [15, 16]. Some researchers proposed that NPE may be resulted, in part, from select pulmonary venoconstriction after massive sympathetic discharge following brain injury. Videlicet, pulmonary venues may be α- and β-adrenergic hypersensitive after brain injury. This could increase ICP and pulmonary pressures, and then induce direct myocyte injuries with wall motion abnormalities [17]. These suggested that vasomotor centers by autonomic nervous system were over-estimated, which could to be an explanation to the association of the edema with central nervous system [18]. However, it could not explain the presence of red blood cells and protein in the alveolar fluid [19, 20]. In 2009, Mascia et al. [21] described the “double hit” model, in which, they observed that systemic inflammatory reaction could induce alteration in blood–brain barrier permeability and promote infiltration of activated neutrophils and macrophages to lung, thus cause direct damage to lung. Cascade of inflammatory response can cause extravasation of intravascular fluid and the damage of blood vessel walls, which can cause intra-alveolar hemorrhage [21, 22]. These observations suggested that a new therapeutic intervention for lung dysfunction is necessary after stroke.

Bone marrow mesenchymal stem cell (BMSCs), as multiple differentiation progenitor cells, are noted to be able to influence native immunomodulatory function [23], suppress local inflammatory response [24] and attenuate sepsis by means of maintaining the normal pulmonary endothelial, establishing epithelial interactions and promoting epithelial function [25, 26]. Therefore, it may be considered as a potential strategy for the treatment of lung injury, and the underlying mechanism needs to be investigated.

In this study, we firstly explored whether BMSCs transplantation could alleviate inflammatory activities and improve functional consequence in lung injury induced by brain ischemia, then investigated the possible molecular mechanism involving in the expression of TNF-α.

## Methods

### Animal and grouping

**Fig. 1:**
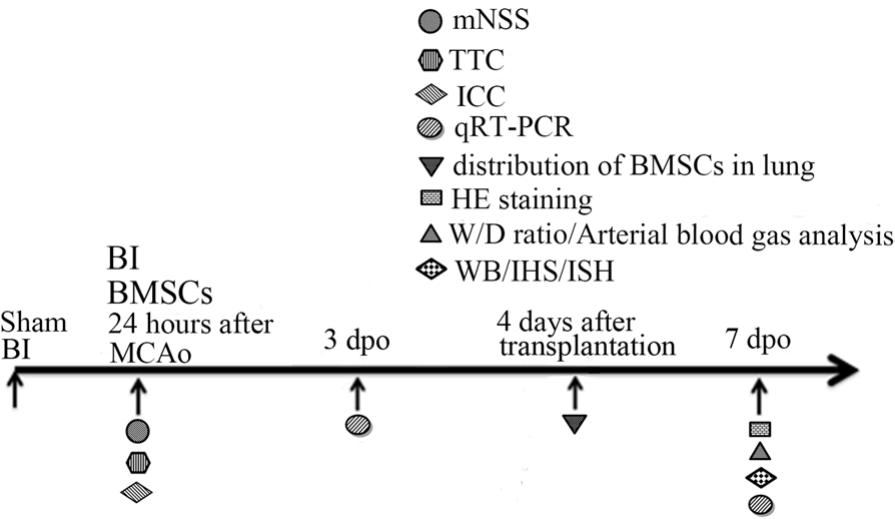
Experiment design for transplantation of BMSCs in rat with brain ischemia-induced pulmonary injury. *mNSS* modified neurological severity score for measuring neural function after brain ischemia. *TTC staining* 2, 3, 5-Triphenyltetrazolium chloride for measuring the viability of brain tissue. *ICC* immunocytochemistry staining to identify the BMSCs. *qRT-PCR* quantitative real-time polymerase chain reaction for detecting the mRNA expression of TNF-α. *HE* *staining* hematoxylin-eosin staining. *W/D ratio* wet-to-dry weight ratio of lung. *WB* western blot analysis for quantifying the protein level of TNF-α. *IHS* immunohistochemistry staining for analyzing the expression of TNFα. *ISH* in situ hybridization for determining the location of TNF-α in lung with cerebral ischemia. *BI* brain ischemia. *BMSCs* brain ischemia with BMSCs transplantation

Forty-five adult male Sprague–Dawley (SD) rats (weighing 180–220 g) were provided by the Center of Experimental Animals, Sichuan University. All animal care, breeding, and testing procedures conform to the principle of *Guidance Suggestions for the Care and Use of Laboratory Animals* promulgated by Ministry of Science and Technology of the People’s Republic of China in 2006, and was approved by the Animal Care and Use Committee, Sichuan, University, Chengdu, China. All animals were housed in individual cages in a room with a temperature of 21–25 °C and a humidity of 45–50 % with a 12 h light/dark cycle and ad libitum access to pellet chow and water. Three groups, 15 rats in each group, were randomly designated as sham group, brain ischemia group (BI) and BMSCs transplantation group (BMSCs), as shown in Fig. 1.

### Induction of focal cerebral ischemia

Permanent focal cerebral infarction was introduced by bipolar coagulation of the left middle cerebral artery (MCA) as described previously [10]. After 3.6 % chloral hydrate (1 ml/100 g) intraperitoneally injection, left common, internal and external carotid arteries were exposed through a midline neck incision and were carefully dissected from the surrounding tissues with help of an operating microscope. After electrocoagulation of the external and common carotid arteries, a 3-0 silicon rubber-coated monofilament (Shadong Biotech, Beijing, China) was inserted through the common carotid artery into the internal carotid artery 18–20 mm beyond the carotid bifurcation to the base of the middle cerebral artery, while 10 mm for sham group. The pterygopalatine branch of the internal carotid artery was exposed before the insertion in order to avoid the filament turning into it. Rectal temperature was maintained at 36.5–37 °C using a heat lamp during the operation and for 2 h after MCAO, and breath and heart rate were monitored all the time.

### Assessment of neurological function

Each rat was subjected to a series of behavioral tests by using modified neurological severity score (mNSS) [27] 24 h after MCAO to identify the model reliability. The mNSS (0–18) is determined by motor (muscle status, abnormal movement), sensory (visual, tactile, proprioceptive), reflex, and balance tests. In the severity score of injury, one score point is awarded for the inability to perform the test or for the lack of a tested reflex; thus, the higher the score is, the more severe the injury is. All rats were given enough time to become familiar with the testing environment before inflicting the brain injury. This test was completed by three trained and qualified observers who were blinded to the groups of animals.

### Isolation of bone marrow mesenchymal stem cell (BMSCs)

BMSCs from SD rats (4 weeks, 60–80 g) were isolated and harvested as described previously [11]. In brief, bone marrow tissues were acquired from the cavities of femurs and tibias with a syringe and 22-gauge needle and injected into the culture medium (Dulbecco’s modified Eagle’s medium, Gibco, Carlsbad, CA, USA; 10 % fetal bovine serum, Hyclone, Logan, UT, USA; 2 mM l-glutamine; 10,000 U/L penicillin, and 10 mg/L streptomycin, Gibco BRL, Life Technologies, Paisley, United Kingdom). All the flushing fluid was turned into the single-cell suspension and seeded into 15 ml culture flasks with culture medium. Cells were cultured at 37 °C in a humidified environment with 5 % CO_2_. Non adherent cells were removed 24 h later, and adherent cell colonies were washed three times with phosphate-buffered saline solution (PBS, Life Technologies). Fresh complete medium was added and changed every 3–4 days. Cells were subcultured 1:2 or 1:4 when they reached 80–90 % confluence. Cells used in this experiment were all harvested from the third passage. Cell surface markers CD29, CD44 and CD45 were detected by immunocytochemistry staining to identify BMSCs.

### Immunocytochemistry staining

Monolayer and single colony-derived adherent cells (at the third passage in culture) were analyzed by immunocytochemistry. Cytospin preparations and growing cells in 6-well culture plate were fixed in 4 % paraformaldehyde for 20 min at 4 °C, washed three times with PBS, and then incubated with 3 % hydrogen-peroxide (H_2_O_2_; Sigma-Aldrich) at room temperature for 30 min. The cells were permeabilized and pre-incubated with blocking solution (containing 2 % goat serum, 0.3 % Triton X-100, and 0.1 %BSA in PBS) for 30 min at room temperature, and then blocked with 5 % normal goat serum at room temperature for 30 min. Washed slides were separately incubated with anti-CD44 (1:50, Abcam, USA), anti-CD29 (1:50, Abcam, USA) and anti-CD45 (1:50, Abcam, USA) primary antibody at 4 °C overnight. After washing with PBS, Alexa Fluor 488 anti-goat IgG (1:200; Molecular Probes, Carlsbad, CA, USA) was incubated for 1 h at 37 °C. Nuclear staining was performed by treatment with 4′, 6-diamidino-2-phenylindole (DAPI, 1:20,000; Molecular Probes, Carlsbad, CA, USA) for 5 min. Slices were then mounted and observed with a florescent microscope (Leica, Solms, Germany).

### BMSCs death assessments of Trypan Blue staining in vitro

Before transplantation, the cell mortality of BMSCs was evaluated by Trypan blue assay (Sigma-Aldrich, St. Louis, MO, USA). BMSCs suspension was balanced in PBS. 500 µL of 0.4 % Trypan Blue solution (w/v) was transferred to a test tube. Then 300 µL of PBS and 200 µL BMSCs suspension (dilution factor = 5) were added and mix thoroughly. Cells were counted using a countess automated cell counter (Invitrogen Life Technologies, Grand Island, NY Life Technologies, Grand Island, NY, USA) after 3 min staining at room temperature. The dead cells were stained with blue color. Count cells on top and left touching middle line of the perimeter of each square. And cells touching the middle line at bottom and right sides were not count. Cell inhibitory ratio was calculated by the following formula: cell mortality (%) = (the dead cell number/the total number) × 100 %.

### BMSCs transplantation procedures

BMSCs from SD rats were prepared and transplanted into SD rats. Two days before and after BMSCs transplantation, SD rats were injected Cyclosporin A (10 mg/kg) for three consecutive days. To trace the BMSCs after transplantation, they were labeled with Hoechst33342 with a final concentration of 10 μM in the culture medium and were incubated for 2 h. BMSCs were washed three times with PBS to remove unbound Hoechst dye, digested with 0.25 % (w/v) trypsin (Gibco), and then suspended in complete medium. After centrifuged and washed with PBS for several times, cells were suspended in a serum-free medium at 1 × 10^6^ cells per 1 ml. In BMSCs group, the rats were given an intravenous (through tail vein) injection of 2 ml of BMSCs suspension with 2 × 10^6^ cells for 10 min one day after MCAO. Penicillin (20 U/rat/day) was injected intraperitoneally for three consecutive days after BMSCs transplantation to prevent infection. The route, dose and timing of administration have been used in a previous study [28–30].

### Tissue preparation

Animals were re-anaesthetized with 3.6 % chloral hydrate (1 ml/100 g). The chest cavity was opened and the hilum of left lung was ligatured. The right lung was perfused with normal saline for 10 min and then slowly fixed with 4 % paraformaldehyde for 30 min. The brain and right lung were obtained and post fixed in 4 % paraformaldehyde (solution in 0.1 mol/L phosphate buffer, pH 7.4) overnight at 4 °C. The sample was then dehydrated in 20 % sucrose solution in 4 % paraformaldehyde, and then with 30 % sucrose solution in 0.1 mol/L phosphate buffer. Subsequently, samples were embedded in paraffin and sectioned (5 μm in thickness). The left lung without fixing was harvested and stored at −80 °C for west blotting. In addition, 3 days after MCAO, lung tissue was also harvested for qRT-PCR. Sections obtained 4 days after BMSCs transplantation were also employed to observe the distribution of Hochest33342 labeled BMSCs lung tissue under fluorescence microscope.

### 2, 3, 5-Triphenyltetrazolium chlorides(TTC) staining and evaluation of infarction volume after MCAO

Viability of brain ischemia was evaluated using TTC (Sigma-Aldrich, St. Louis, Missouri, USA) 24 h after MCAO. Rat brains were rapidly removed, frozen at −80 °C for 5 min and then sliced coronal into serial 2-mm-thick slices at the level of the bregma. Sets of five serial slices from each brain were incubated for 30 min at 37 °C in 2 % TTC (Sigma Co., St Louis, MO, USA) in the dark washed in PBS, and then fixed by 4 % formaldehyde in PBS. Images of sections from the exact center of the forebrain were captured using digital camera system (Leica, Solms, Germany). The infarction area and hemisphere area of each section were traced and measured using Image Pro plus 6.0 software (Media Cybernetics, Inc., MD, USA). To eliminate the interference of brain edema, the infarct volume was corrected by standard methods (contralateral hemisphere volume–volume of non-ischemic ipsilateral hemisphere). Infracted volume was expressed as a percentage of the contralateral hemisphere, as described previously [31, 32].

### Lung function assessment and edema measurement

Arterial blood gas analysis and wet-to-dry weight (W/D) ratio of lung were used to represent the severity of lung edema. Seven days after MCAO, rats were re-anaesthetized with 3.6 % chloral hydrate (2 ml/100 g) intraperitoneally. Arterial blood samples (100 μl) of rats were extracted into a heparinized syringe by left ventricular sampling, and then blood gas analysis was immediately performed with blood-gas analyzer (RADUOMETER ABL800). In addition, after equivalent lung tissue in bilateral was separated from the thoracic cavity, the lungs were weighed and then dried to constant weight at 80 °C for 24 h. The ratio of wet-to-dry was finally calculated using the formula of (dry weight/wet weight) × 100 %.

### Hematoxylin–eosin staining and immunohistochemical assay

Brains and lung were removed and placed in 10 % paraformaldehyde in phosphate-buffer overnight, dehydrated, and embedded in paraffin. 5 μm-thick serial sections of the right lung and brain were stained with hematoxylin-eosin (HE). For immunohistochemical assay, the paraffin embedded sections were routinely de-paraffinized and rehydrated. After non-specific antigen site blocking, the slices of lung tissue were incubated with monoclonal mouse anti-TNF-α (dilution 1:50, Abcam, Cambridge, UK), and then the slices were performed at room temperature for 1 h and then incubated overnight at 4 °C. The sections were incubated in biotin-conjugated secondary antibody for 30 min at 37 °C and then incubated with horseradish peroxidase conjugated streptavidin avidin for 20 min at 37 °C. Washing was performed with PBS (pH 7.4) for three times (5 min for each time) between any two adjacent procedures, except for blocking in serum. Then, visualization was done with diaminobenzidine (DAB), followed by dehydration, transparentization and mounting. Counterstaining of sections by hematoxylin was also performed. Negative controls were stained similarly. However, PBS was used, instead of a primary antibody. Images were taken with a laser scanning confocal microscope (Nikon, Tokyo, Japan). Quantification of the immunolabeled lung sections was performed separately. For each slice, ×200 magnification photomicrographs were taken to measure the density of TNF-α. The mean density was presented as IOD over the area of interest using Image-Pro plus 6.0 software (Media Cybernetics, Silver Spring, MD, USA), which was described previously [18]. Data are presented as mean ± SEM. Observers were blinded to group identity.

### In situ hybridization

Slides, prepared as described, were used for ISH. Briefly, sections were de-waxed in xylene, rehydrated in gradedalcohols, and placed in diethyl pyrocarbonate (DEPC) H_2_O. Endogenous peroxidase was inactivated by incubation in 3 % H_2_O_2_ for 15 min at room temperature. Sections were then digested in proteinase K (20 μg/ml) for 20 min, rinsed in NaCl/Tris, and then fixed in 4 % PFA for 10 min. Following this, slides were rinsed with PBS twice for 5 min. Slices were blocked at room temperature for 2 h in hybridization buffer (50 % formamide, 25 % 5× saline sodium citrate (SSC), 10 % 5× Denhardt’s and 15 % DEPC-H_2_O (containing 200 ng/ml yeast RNA, 500 g/ml salmon sperm DNA and 20 mg/ml Roche blocking reagent)), hybridized with 30 nmol of locked nucleic acid (LNA)-modified oligonucleotide probe (Exiqon, Woburn, MA, USA) complementary to TNF-α, and then labeled with digoxigenin (DIG) at 52 °C overnight. After hybridization, the slides were washed in 2× SSC twice at 37 °C for 15 min after washing with 0.5× SSC (15 min at 37 °C) and 0.2× SSC (15 min at 37 °C). The slides were then incubated with HRP conjugated anti-DIG antibody. Sections were rinsed in PBS three times for 5 min. Peroxidase staining was visualized with DAB for 3 min.

### Detection of BMSCs cells and phenotypic analysis in vivo

Four days after transplantation, the phenotype of BMSCs in vivo was detected. Lungs of the deeply-anesthetized rats were removed, fixed in 4 % paraformaldehyde in phosphate-buffer, dehydrated with 30 % sucrose in 0.1 M PBS for overnight, and frozen in powdered dry ice. Cryostat sections (10 μm) were processed. Phenotypic analysis of the transplanted cells in vivo was carried out using fluorescence microscope (Leica, Solms, Germany).

### Quantitative real-time polymerase chain reaction (qRT-PCR)

Three and seven days after MCAO, the mRNA expressions of TNF-α and β-actin in the lung were detected by qRT-PCR. In brief, the left upper lung tissues were kept at −80 °C, then the total RNA was isolated from lung homogenates with Trizol reagent (Takara Bio Inc., Otsu, Japan) and reverse transcribed using PrimerScript^®^ RT reagent Kit with cDNA eraser (Takara Bio Inc., Otsu, Japan). Each PCR was performed with a iQ™5Multicolor RealTime PCR Detection System (Bio-Rad Laboratories, Inc., USA) and a SYBR Green PCR kit (Takara Bio Inc., Otsu, Japan) in a final volume of 20 μL, containing 1.6 μL cDNA template, forward and backward primers 0.8 μL each, 10 μL SYBR^®^ Premix Ex Taq™II and 6.8 μL dH2O. The primers and Taqman probes were designed using Primer Premier (PREMIER Biosoft International, Canada). The premier sequences were as follows: TNF-α (forward) 5′-AATGACCCAGATTATGTTTGAGAC-C-3′ and (reverse) 5′-TCCAGAGTCCA GCACAATACCAG-3′; β-actin (forward) 5′-GATTACTGCTCTGGCTCCTAGC-3′ and (reverse) 5′-ACTCATCGTACTCCTGCTTGCT-3′. The mouse β-actin housekeeping gene was used as an internal control. The fluorescence emitted by the reporter dye was detected in real time, and the threshold cycle (Ct) of each sample was recorded as a quantitative measure of the amount of PCR product in the sample. The target signal was normalized against the relative quantity of β-actin and expressed as ΔCt = Ct_target_ − Ct_β-actin_. The changes in target signal relative to the total amount of genomic DNA were expressed as ΔΔCt = ΔCt_treatment_ − ΔCt_control_. Relative changes in metastasis were then calculated as 2^−ΔΔCt^ [13].

### Western blot analysis

Seven days after MCAO, the protein expression of TNF-α was determined by western blot. The left middle lung tissues were kept at −80 °C and homogenized in PBS containing the protease inhibitor cocktail with the aid of a tissue grinder. The homogenates were centrifuged for 15 min at 12,000 rpm and 4 °C. Supernatants were collected, and the protein concentration of each sample was measured with Bradford protein assay kit (BioRad Laboratories, Inc., USA) using bovine serum albumin (BSA) as the standard. An equal amount of protein from each sample (25 μg) was resolved in 15 % SDS-polyacryamide gel (SDS-PAGE). After electrophoresis, the separated protein bands were wet transferred to the polyvinylidenedifluoride (PVDF) membrane (Millipore, Bedford, MA, USA). Nonspecific binding to membrane was blocked with 5 % skim milk (Sigma, USA) in TBST (10 mmol/L Tris, pH 8.0,150 mmol/L NaCl, 0.05 % Tween 20) for 1 h at room temperature. The membranes were incubated for 24 h at 4 °C with primary anti-TNF-α IgG (1:1000, Abcam, Cambridge, UK),and anti-β-actin antibody(1:2500, Santa Cruz Biotechnology, Inc., USA) respectively. The secondary antibody (horseradish peroxidase-conjugated goat anti-mouse IgG in 5 % skim milk-TBS-T) was added at 1:20,000 dilutions and incubated for 2 h at room temperature. Immunodetected proteins were visualized using ECL assay kit (Millipore, Bedford, MA, USA) with glyco doc imaging system (Bio-Rad Laboratories, Inc., USA) and analyzed by Quantity One software. The relative protein level was normalized to β-actin.

### Statistic analysis

Sigma plot software SPSS version 17.0 (SPSS Inc., Chicago, USA) was used to perform data analysis. All data were expressed as mean ± standard deviation (SD). Differences between control and experimental group were compared by One-Way analysis of variance (ANOVA) and P < 0.05 was considered to be statistically significant.

## Results

### Cerebral ischemia induced acute lung injury

**Fig. 2:**
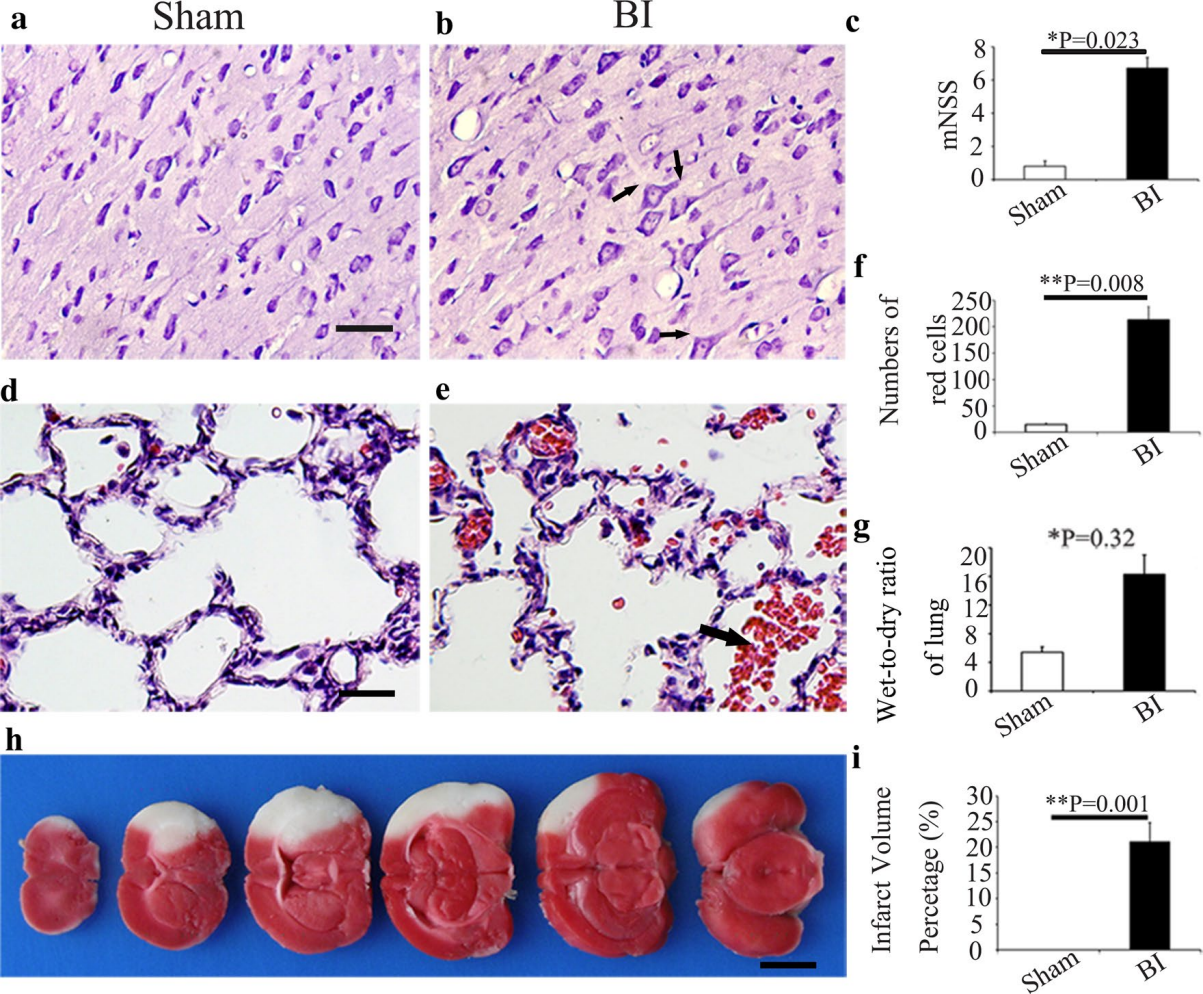
Cerebral ischemia induced by MCAO produces lung injury in rat. **a, b** Morphological examination of the cerebral cortex neurons by H&E staining. **a** Sham group; **b** BI group. **a** In Sham group, there were abundant pyramidal cells. **b** After brain ischemia, the nuclei of pyramidal cells (*bold arrow*) were shrunken and surrounded with acidophilic cytoplasm. **c** mNSS score in the Sham group and BI group at 24 h after MCAO. **d, e** H&E staining of the lung tissue in Sham group (**d**) and BI group (**e**). Red blood cells (*bold arrow*) were observed in the pulmonary alveoli (**e**). **f** *Bar graph* of the numbers of red cells in the lung in Sham group and BI group was shown. **g** Wet-to-dry weight (W/D) ratio of lung in Sham group and BI group was shown. **h** Viability of brain tissue was shown by TTC staining in rats suffered MCAO. **i** *Bar graph* of the infarct volume percentage in Sham group and BI group were shown. *P < 0.05;**P < 0.01, n = 5 in each group. *Scale bars* **a,b, d, e**, 25 μm; **h**, 200 μm

MCAO has a high mortality, about 60–70 %, in our study. After brain ischemia, the pyramidal cells in the cortex were exhibited acellular edema (Fig. 2a, b). Neurologic function evaluation showed that mNSS score was higher in the brain ischemia group 24 h after MCAO, as compared with the Sham group (P < 0.05) (Fig. 2c). Normal lung tissue structures and clear alveoli existed in rats of Sham group, and there are few inflammatory cells in the lung tissue (Fig. 2d). Comparatively, in brain ischemia group, many cells with nuclei were large and deep dyed were observed, and these cells were considered as macrophages (Fig. 2e). The alveolar walls are thickened; capillaries in the alveolar walls are congested with many red blood cells, local hemorrhage, interstitial edema, alveoli exudation and inflammatory cells were observed in lungs in brain ischemia group. Some inflammatory cells were passing through the vascular wall to the pulmonary interstitial. Various pathological changes were observed in the same pulmonary lobe (Fig. 2e). In the brain ischemia group, the counts of red cells in lung were significant increased, as compared with the Sham group (Fig. 2f). Moreover, 7 days after MCAO, wet-to-dry weight (W/D) ratio of lung in brain ischemia group elevated, as compared with the Sham group (P < 0.05, Fig. 2g). TTC staining showed the volume of infarct in the brain of MCAO is apparent as compared with Sham one (Fig. 2h). Ischemic infarcts include ipsilateral frontal lobe, parietal lobe and the front of the temporal lobe after MCAO (Fig. 2h), and the average infarct percentage is approximately 21 %.

### Analysis of lung function and TNF-α expression after brain ischemia

**Fig. 3:**
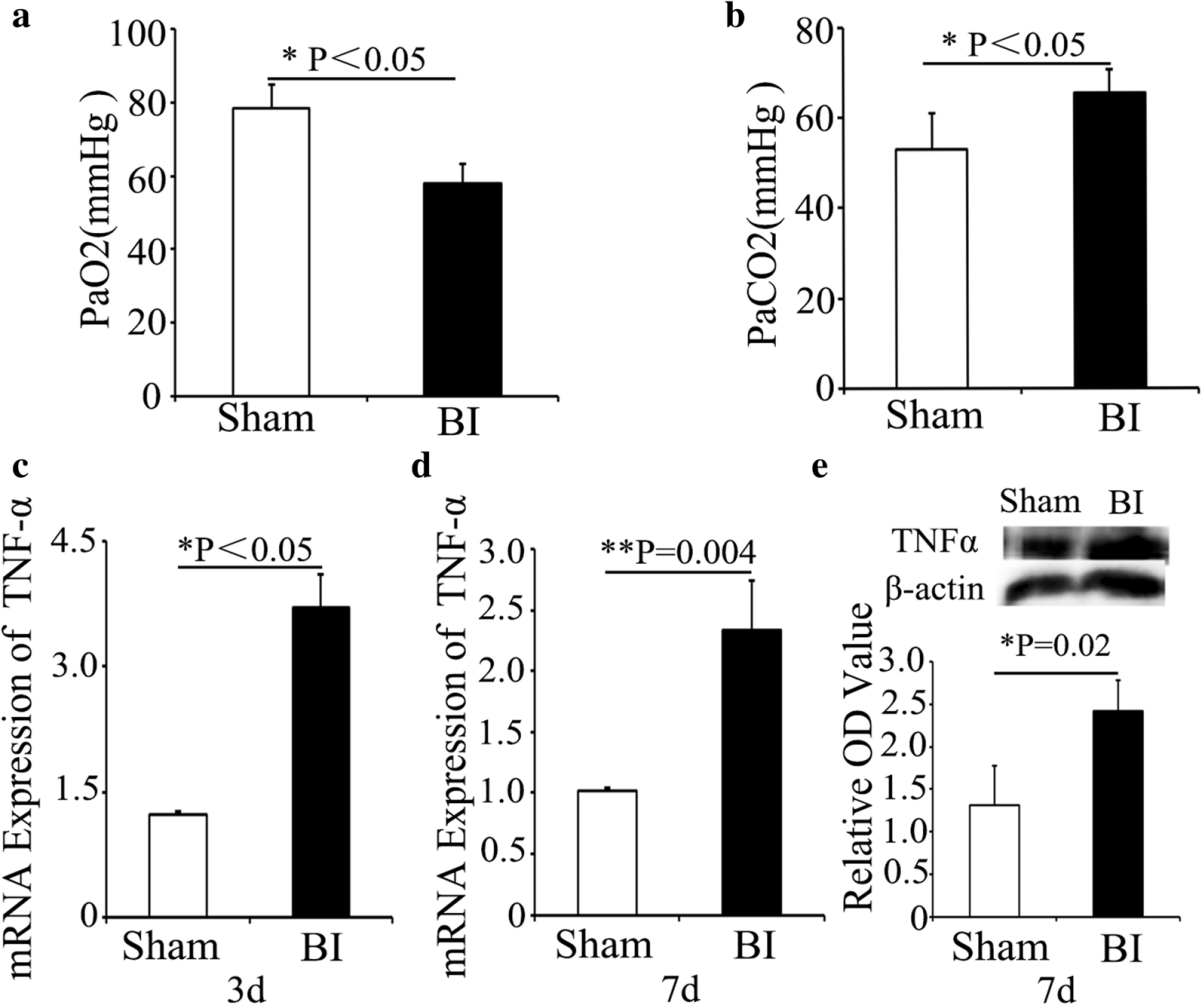
Lung function was deteriorated and the expression of TNF-α in lung was increased after MCAO. **a, b** Blood gas analysis of the Sham group and BI group. PaO2 (**a**); PaCO2 (**b**). **c**, **d** qRT-PCR assessment of the mRNA expression of TNF-α in the Sham group and BI group at 3(**c**) and 7(**d**) days after MCAO. **e** Western blot assessment of the protein expression of TNF-α in lung tissues in the Sham group and BI group. A semi-quantitative analysis was used to represent the total protein level of TNF-α. Data are expressed as mean ± SD. *P < 0.05, **P < 0.01 compared to the Sham group, n = 5 in each group

Blood gas analysis confirmed that PaO_2_ decreased and PaCO_2_ increased after brain ischemia, compared with the Sham group(both P < 0.05, Fig. 3a, b). Either mRNA (Fig. 3c, d) or protein (Fig. 3e) expression of TNF-α in lung significantly increased after MCAO, compared with the Sham group (P < 0.05).

### Expression and localization of TNF-α in lung after brain ischemia

**Fig. 4:**
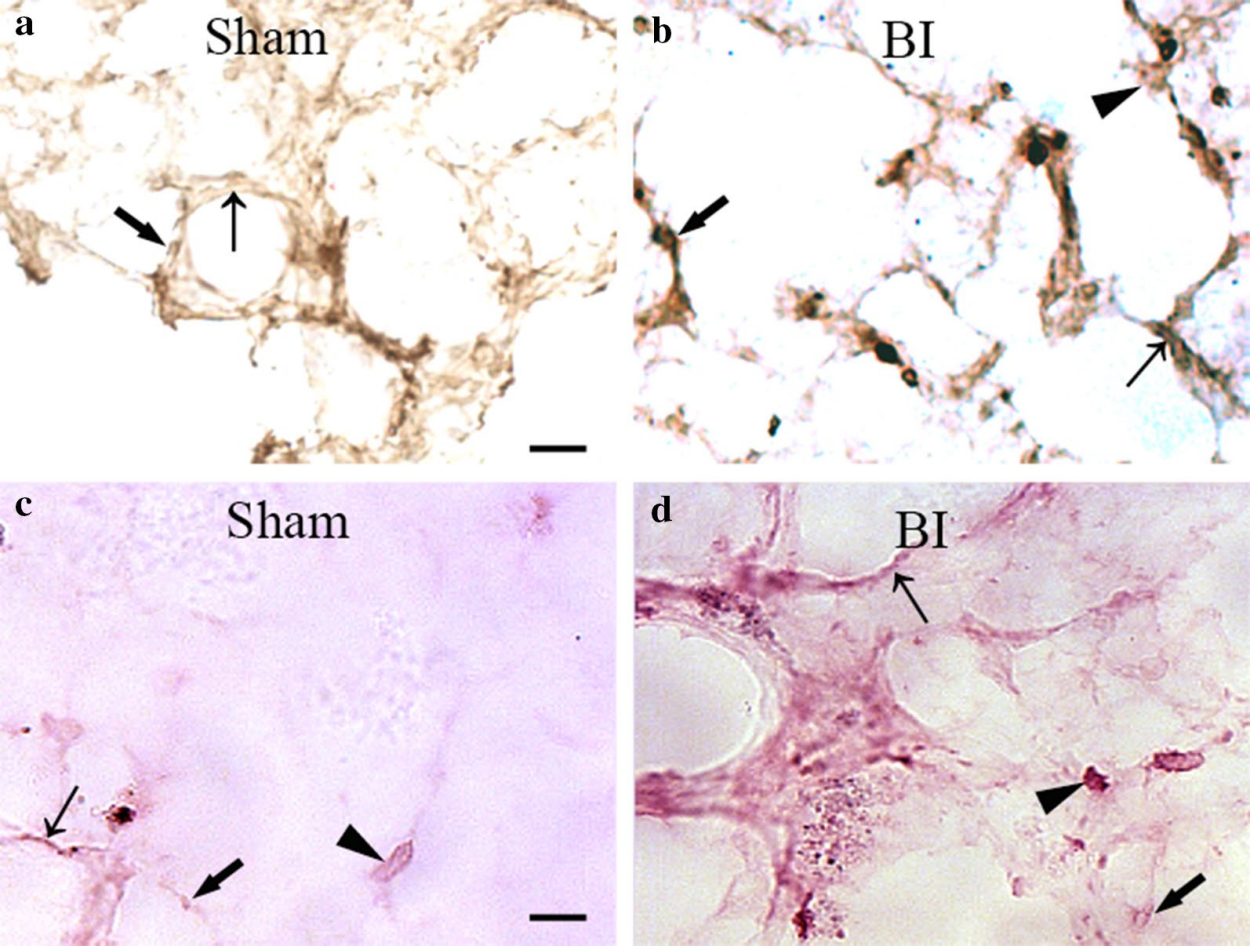
Representative histopathological finding of TNF-α in the injured lung after brain ischemia. **a**, **b** Immunohistochemistry staining of TNF-α in type I (*brown signal, bold arrow*), II lung epithelial cells (*brown signal, thin arrow*) and macrophagocytes (*brown signal, arrow head*) in the Sham group (**a**) and BI group (**b**). **c**, **d** ISH of TNF-α (*red signals*) was detected in the typeI (ISH, *red signals, bold arrow*), II lung epithelial cells (ISH, *red signals, thin arrow*) and macrophagocytes (ISH, *red signals, arrow head*) in the Sham group (**c**) and BI group (**d**). *Scale bars* **a**–**d**, 25 μm

Immunohistochemistry showed that in Sham group, TNF-α staining was junior in alveolar type I and type II epithelial cell, and few macrophagocytes was stained by TNF-α recognition (Fig. 4a). While in brain ischemia group, intensity of staining in typeIand II epithelial cells were both increased, a stronger dyeing of TNF-α was observed in alveolar type I and type II epithelial cells (Fig. 4b). What’s more important, more macrophagocytes with TNF-α strong positive staining were observed in lung after MCAO (Fig. 4b). ISH indicated that TNF-α was mainly located in macrophagocytes, which were significantly increased in the brain ischemia group (Fig. 4c, d).

### Morphological and surface antigen characteristics of primary BMSCs

**Fig. 5:**
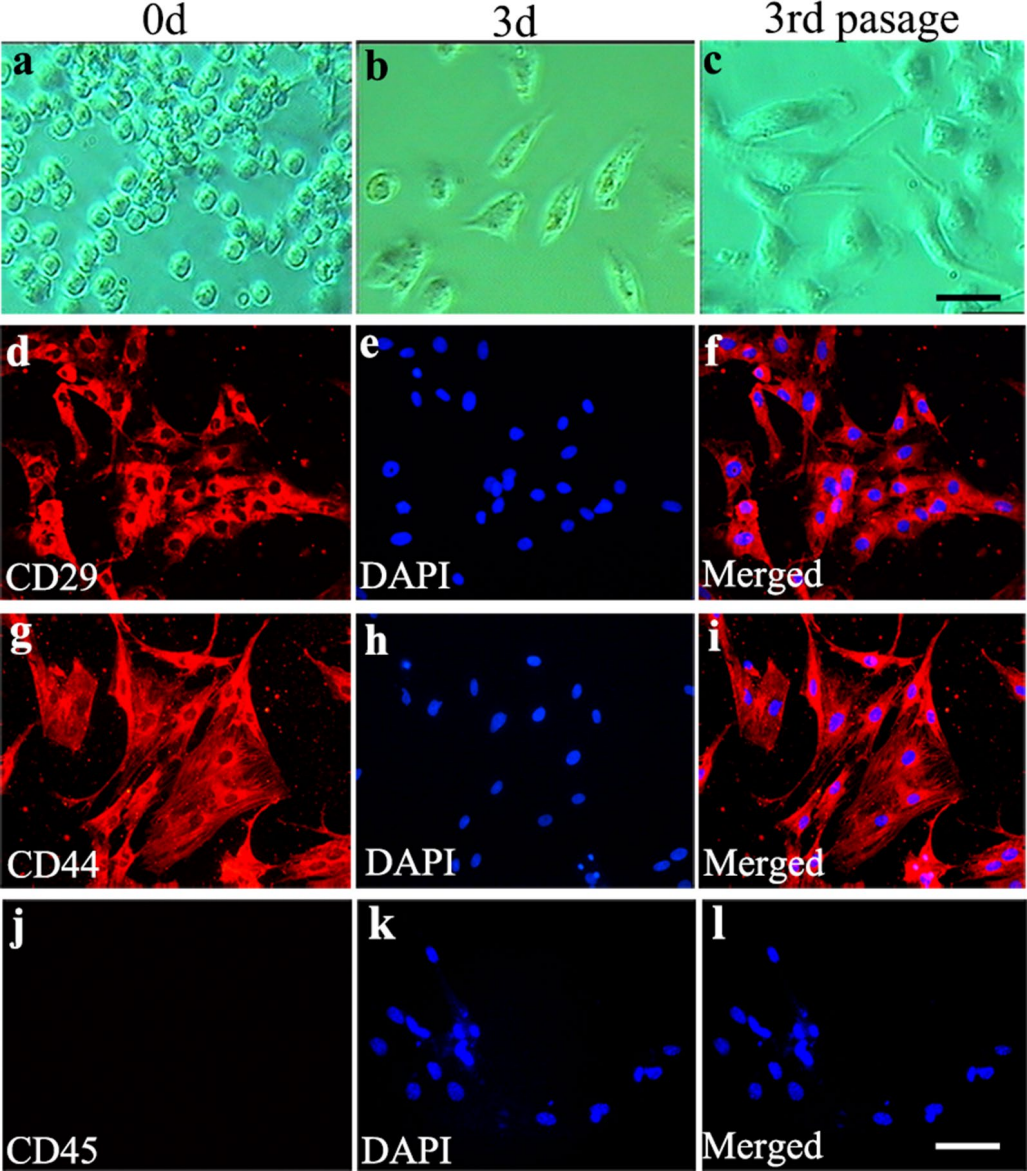
Isolation and identification of BMSCs. **a**, **b**, **c** Cellular morphology of BMSCs at 0 h (**a**) 3-day (**b**) and the 3rd passage (**c**) in primary culture which were observed under inverted phase contrast microscope CD29, CD44 and CD45 immunostaining of the 3rd passage of BMSCs were performed. Immunostaining of CD29 (**d**, *red*), CD44 (**g**, *red*) and CD45 (**j**) with nucleus stained DAPI (**e**, **h**, **k** respectively, *blue*) and the merge graphs (**f**, **i**, **l**) were showed. *Scale bars* **a**, **b**, **c**, 100 μm; **d**–**l**, 25 μm

Primary BMSCs cultured as plastic adherent cells. They turned to confluency one week after culture and were expanded by serial subcultivation just prior to confluency. Figure 5a showed the morphological features of BMSCs at 0 h. At the 3rd day, their clonal-rosette derived round shape became elongated or spindle-shaped (Fig. 5b). At the 3rd passage, the form of primary BMSCs adherent cells was uniform with polygons-shaped appearance (Fig. 5c). To characterize primary BMSCs phenotype, we examined the cell surface markers CD29, CD44, and CD45 by means of immunocytochemistry staining. The BMSCs were positive for CD29 (Fig. 5d–f) and CD44 (Fig. 5g–i), but negative for CD45 (Fig. 5j–l). This indicates that the cells were highly expressed markers of mesenchymal stem cells and were negative for endothelial and hematopoietic cell markers.

### BMSCs engrafts improved lung morphology and alleviated TNF-α expression

**Fig. 6:**
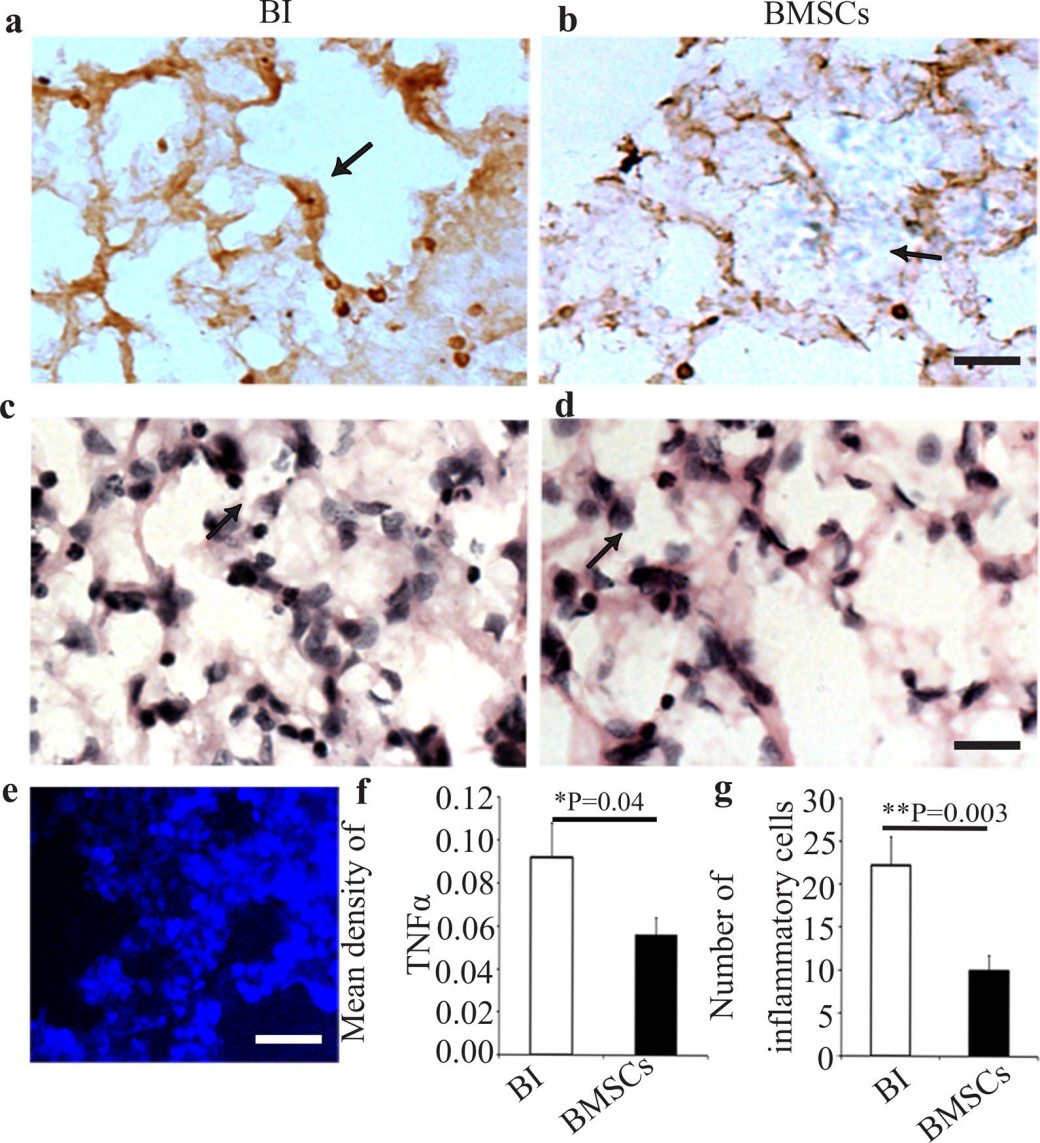
BMSCs transplantation decreased the expression of TNF-α and the numbers of inflammatory cells in lungs of rats after MCAO. **a**, **b** The intracellular TNF-α in lung (*brown signal, thin arrow*) was detected by immunohistochemistry. The photos represented the intracellular TNF-α content of lung in BI group (**a**) and BMSCs group (**b**). **c**, **d** Inflammatory cells in lung of rats in BI group (**c**) and BMSCs group (**d**) were detected by HE staining. **e** Hochest33342 labeled cells (*blue*) in lung under fluorescence microscope. **f** *Bar graph* of mean density of TNF-α in the lung in BI group and BMSCs group. **g** Numbers of inflammatory cells were calculated in BI group and BMSCs group. (*P < 0.05, n = 5 in each group). **a**–**d**, 25 μm; **e**, 100 μm

Trypan Blue staining indicated that the viability of BMSCs is about 96 %, before transplantation. Brain ischemia induced prominent lesions in lung and increased the inflammatory exudations in alveolar and inflammatory cells (Fig. 6a, c). In contrast, inflammatory exudations in alveolar and inflammatory cells were partly ameliorated by BMSCs treatment (Fig. 6b, d). Within the lung tissue, the transplanted BMSCs could be easily identified under fluorescent microscopy, and their nucleic appearances were exactly the same as that before the transplantation (Fig. 6e). Cells derived from BMSCs were round-to-oval with irregular dark brown nuclei and thin cytoplasm by Hoechst33342 staining (Fig. 6e). Moreover, both the mean density of TNF-α (Fig. 6f) and the number of the inflammation cells (Fig. 6g) were significantly decreased in BMSCs transplantation group (vs brain ischemia group, P < 0.05).

### Analysis of lung function and TNF-α expression after treatment of BMSCs in brain ischemia rats

**Fig. 7:**
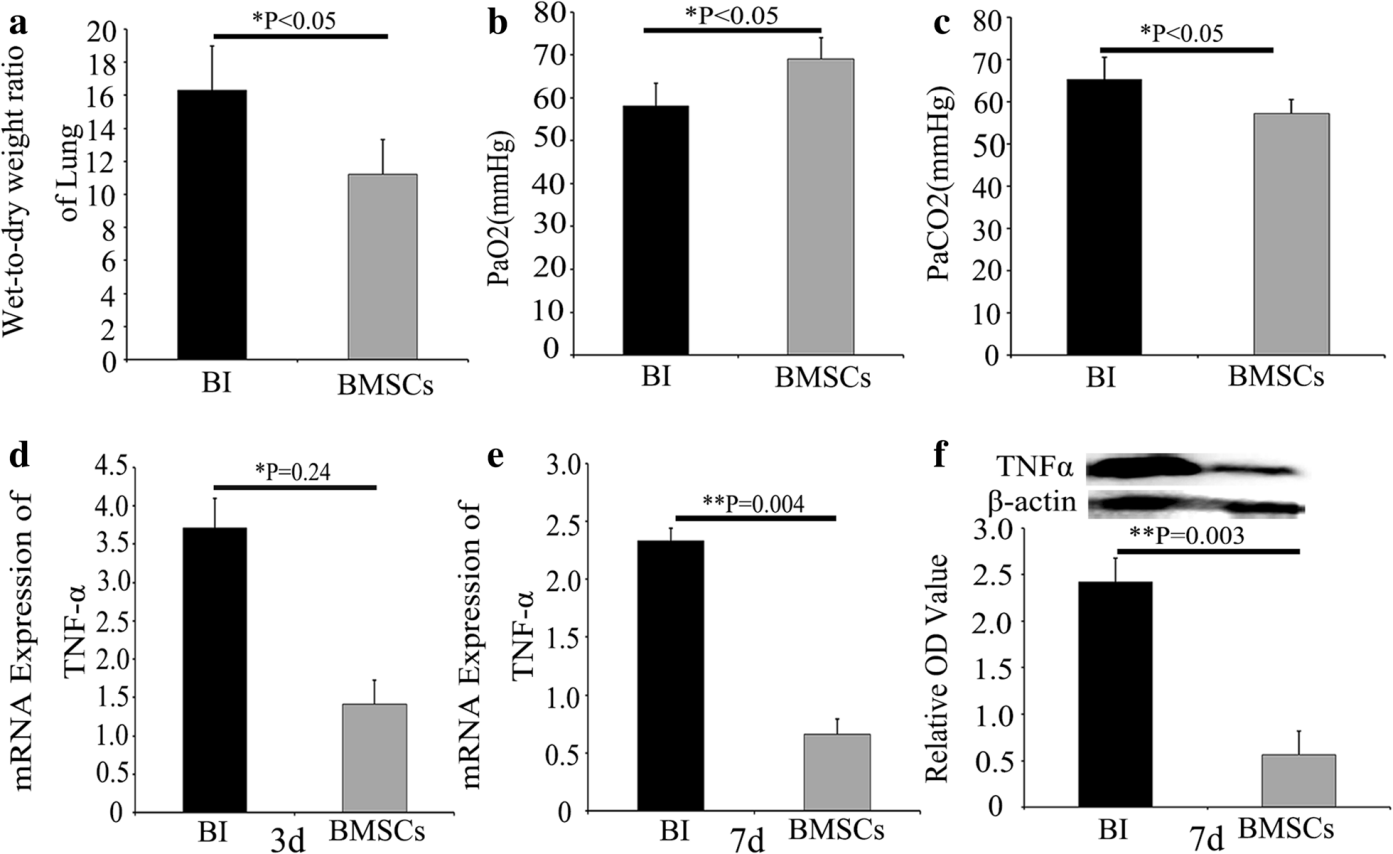
BMSCs ameliorated lung function and reduced the expression of TNF-α in lung after MCAO. **a** Wet-to-dry weight (W/D) ratio of lung in BI group and BMSCs group. **b**, **c** Blood gas analysis of the BI group and BMSCs group. PaO2 (**b**); PaCO2 (**c**). **d**, **e** mRNA expression of TNF-α in BI group and BMSCs group at 3 (**d**) and 7 (**e**) days after MCAO. **f** Western blot assessment of the protein expression of TNF-α in lung tissues in BI group and BMSCs group 7 days after MCAO. Semi-quantitative analysis was used to represent the total protein level of TNF-α. Data are expressed as mean ± S.D. *P < 0.05, **P < 0.01 compared to the BI group, n = 5 in each group

Seven days after BMSC transplantation, wet-to-dry weight(W/D) ratio of lung in BMSCs group decreased, as compared with rats subjected to only brain ischemia (P < 0.05, Fig. 7a). Blood gas analysis indicated that PaO_2_ increased (P < 0.05, Fig. 7b) and PaCO_2_ decreased (P < 0.05) (Fig. 7c) after BMSCs treatment, as compared with the brain ischemia group. Furthermore, there was a significantly decrease in the level of TNF-α (Fig. 7d, e) mRNA or protein expression (Fig. 7f) both 3 and 7 days after BMSCs transplantation, as compared with the brain ischemia group (both P < 0.05).

## Discussion

The major findings of these experiments can be summarized as follows: (1) brain ischemia could induce lung injury, a kind of inflammatory lung damage; (2) intravenous injection of BMSCs could home to the lung and survive in brain ischemia induced lung injury model; (3) treatment with BMSCs could reduce pulmonary edema, improve lung function after brain ischemia; (4) BMSCs exhibited a positive role in suppressing inflammation mediators like TNF-α in lung after brain ischemia.

In this study, we found that permanent MCAO induced pulmonary injury in SD rats. Pulmonary dysfunction is an important independent factor that affects mortality in patients suffering brain injury and deteriorates the long-term neurologic outcome [13, 32–34]. Ségolène Mrozek et al. suggested that brain lung crosstalk is a complex interaction from the brain to the lung but also from the lung to the brain. Several studies described the occurrence of severe pulmonary injuries after experiencing a brain injury, such as severe traumatic brain injury, subarachnoid hemorrhage, stroke or hypoxic-ischemic brain damage [8, 10, 33–35]. The pathophysiology of brain-lung interaction is complex and several hypotheses have been proposed with a particular described “double hit” model [21]. After brain ischemia, there was local hemorrhage, interstitial edema, alveoli exudation and inflammatory infiltrates in lung. This injury presents as a kind of inflammatory lung damage characterized by histology study and increased tissue TNF-α concentration in the lung.

Intravenous injection of BMSCs could home to the lung and survive after brain ischemia. The ability to self-renew and multi-directional differentiation make BMSCs attractive as a potential treatment for acute lung injury. Four days after BMSCs treatment, migration towards and adhesion of BMSCs with Hochest33342 labeled were observed in the rat lung tissue under fluorescence microscope Muhammad Aslam et al. [23] did observe that a higher number of donor BMSCs can be detected in the injured lung compared with the normal lung at 10 days post injection. This elucidated that the intravenous transplanted BMSCs could partially maintain themselves in the injured lung. Although we cannot propose that BMSCs extensively replace injured lung cells to effectively improve lung architecture because of the minimal BMSC engraftment after transplantation, this may be the basis for functional and morphological improvement [23, 36].

In the present study, we have shown that BMSCs enhance the recovery of lung structure and function after brain ischemia. Specifically, BMSCs treatment improved lung architecture, restored static lung compliance, and ameliorate alveolar fluid exudation in lung, as evidenced by reduced lung wet- to- dry weight ratio, histological evidence of reduced alveolar tissue edema and the improved blood gas analysis. These findings support previous findings that BMSCs normalize lung fluid balance and alveolar fluid clearance through therapeutic effects on the lung endothelium [37]. The integrity of the lung microvascular endothelium is essential to prevent the influx of protein-rich fluid from the plasma as well as inflammatory cells which may further aggravate the ability of the lung epithelium to remove alveolar edema.

BMSCs suppress inflammation responses in lung after brain ischemia. BMSCs were demonstrated by prior studies to have anti-inflammatory effects on lung injury. Gupta et al. [11] found that intrapulmonary delivery of BMSCs in mice mediated a down-regulation of pro-inflammatory responses to endotoxin by reducing TNF-α and macrophage inflammatory protein-2, while increasing the anti-inflammatory cytokine IL-10. BMSCs therapy can attenuate paraquat-induced ALI in rats through decreases plasma TNF-α and MDA levels. BMSCs have demonstrated benefit in oleic acid induced acute lung injury in rats by decreased TNF-α expression and increased IL-10 content [38]. BMSCs decreased lung inflammation ventilator-induced lung injury rat, by reduced TNF-α and upregulated IL-10 [39].

In our study, we found a significantly increase of TNF-α concentration in the lung tissue after brain ischemia. Inflammatory cytokines may key mediator for pulmonary alveoli injury. TNF-α could distract vascular endothelial cells, leading to the increase of capillary permeability and the development of lung inflammation response [40]. TNF-α induced NF-κB signaling activation in whole lung has been proven to be associated with inflammation damage in pulmonary complication [41, 42].

On the other hand, it’s generally accepted that the beneficial effects of stem and progenitor cells in animal disease models are the result of immunomodulatory and trophic support properties delivered by the transplanted cells acting in a paracrine manner. BMSCs might produce a number of potent cytokines and growth factors, such as platelet-derived growth factor (PDGF) and transforming growth factor β (TGF-β1) to repair the process of lung injury [43, 44].

We performed the cell transplantation at 24 h after stroke, which needs to be justified. It was reported that BMSC transplantation acutely after stroke was deteriorating to the ischemic brain. The high level of VEGF in these cells may cause brain edema. However, the relationship between VEGF and brain edema has been debated, also. Some literatures demonstrated that the up-regulated VEGF after ischemic stroke may significantly increase the brain water content [45–47] and induce vasogenic brain edema [48, 49]. VEGF has the potency to increase vascular permeability [50, 51]. After brain ischemia, VEGF could significantly increase BBB leakage [52]. VEGF disrupts the organization of interendothelial junctions (IEJ) and the integrin-extracellular matrix (ECM) complexes, thereby opening the junctional barrier. Nicholas van Bruggen et al. [53] established that sequesters murine VEGF could significantly reduce the acute appearance of cortical edema after focal cerebral ischemia [53]. However, the role of VEGF in the pathogenesis of the formation of ischemic brain edema is unclear with contradictory experimental observations. For example, Hayashi et al. reported that VEGF itself, when applied topically to the surface of a reperfused rat brain after transient cerebral artery occlusion, reduced ischemic brain damage, infarct volume, and edema formation. Harrigan MR et al. [54] demonstrated that intracerebroventricular infusion of VEGF decreases infarct volume and brain edema after temporary MCAO. Betz et al. [55] suggested that ischemic brain edema formation in the relatively early phase is mainly caused by cytotoxic edema rather than vasogenic edema [56]. On the other hand, VEGF may protect the blood–brain barrier rather than destroy it after brain ischemia because Criscuolo et al. suggested that VEGF application through the carotid artery did not cause albumin extravasation. The amelioration of blood–brain barrier injury may be attributable to the protective effect of VEGF on endothelial cells [57]. One possible mechanism of VEGF in the reduction of ischemic brain edema is that VEGF could reduce the infarct volume by protection of endothelial cells, and thus reduce water content, without exacerbating vasogenic edema. Chen Bo et al. [54] suggested that VEGF gene modified BMSCs adenovirus could reduce reactive gliosis, ameliorate neurological deficit, diminish the percentage of cerebral infarction volume in rats, and facilitate angiogenesis [58]. In addition, BMSCs transplantation could ameliorate neurological deficit and increase neurogenesis as the result of VEGF-mediated angiogenesis without brain ischemia [59]. Therefore, it seems that BMSCs combined with VEGF may play a more significant role in protecting brain ischemia.

Together, our results give vital evidences that BMSCs treatment may be a potential effective therapy for pulmonary complication after brain ischemia derived brain ischemia. Moreover, BMSCs may regulate TNF-α expression in brain ischemia induced lung injury. Therefore, BMSCs treatment can reduce lung injury, in which, the possible mechanism may link to the inhibition of TNF-α in brain ischemia induced lung injured rats. Our findings is first time to show the protective effects of BMSCs in brain ischemia induced lung injury, which is linked to the inhibition of TNF-α expression.

## Conclusions

We concluded that the intravenous transplantation of BMSCs originated from the bone marrow could partially ameliorate lung injury induced by brain ischemia, and inhibited the mobilization of inflammatory cells and reduce the TNF-α protein expression in lung. The present data indicate that BMSCs may serve as the basis of a novel therapeutic approach for patients suffering from pulmonary complications after brain injury, based on the TNF-α inhibition, in future clinic practice.
